# Decoding Seafood: Multi-Marker Metabarcoding for Authenticating Processed Seafood

**DOI:** 10.3390/foods13152382

**Published:** 2024-07-27

**Authors:** Anna Mottola, Roberta Piredda, Lucilia Lorusso, Lucia Ranieri, Chiara Intermite, Concettina Barresi, Carmela Galli, Angela Di Pinto

**Affiliations:** 1 Department of Veterinary Medicine, University of Bari Aldo Moro, Prov. le Casamassima 62, Km 3, 70010 Valenzano, Italy; anna.mottola@uniba.it (A.M.); robpiredda@gmail.com (R.P.); lucia.ranieri@uniba.it (L.R.); chiara.intermite@studenti.unisalento.it (C.I.); angela.dipinto@uniba.it (A.D.P.); 2 Laboratory of Modena, Department of Central Inspectorate for Fraud Repression and Quality Protection of the Agri-Food Products and Foodstuffs, Ministry of Agriculture, Food Sovereignty and Forests (ICQRF-MASAF), Via Domenico Cucchiari, 12, 41124 Modena, Italy; c.barresi@masaf.gov.it; 3 Laboratory of Salerno, Department of Central Inspectorate for Fraud Repression and Quality Protection of the Agri-Food Products and Foodstuffs, Ministry of Agriculture, Food Sovereignty and Forests (ICQRF-MASAF), Via Frà Giacomo Acquaviva n. 1, 84135 Salerno, Italy; c.galli@masaf.gov.it

**Keywords:** seafood traceability, multi-species products, NGS, dual-marker, primers, food official controls

## Abstract

Given the recognized nutritional value of fish and shifting consumer lifestyles, processed seafood has become increasingly prevalent, comprising a significant portion of global food production. Although current European Union labeling regulations do not require species declaration for these products, food business operators often voluntarily provide this information on ingredient lists. Next Generation Sequencing (NGS) approaches are currently the most effective methods for verifying the accuracy of species declarations on processed seafood labels. This study examined the species composition of 20 processed seafood products, each labeled as containing a single species, using two DNA metabarcoding markers targeting the mitochondrial cytochrome c oxidase I (COI) and 16S rRNA genes. The combined use of these markers revealed that the majority of the products contained multiple species. Furthermore, two products were found to be mislabeled, as the declared species were not detected. These findings underscore that NGS is a robust technique that could be adopted to support routine food industry activities and official control programs, thereby enhancing the ‘From Boat to Plate’ strategy and combating fraudulent practices in the complex fisheries supply chain.

## 1. Introduction

Seafood has emerged as one of the most highly traded food commodities globally, driven by its relatively low cost and recognized nutritional importance in the human diet [1,2]. Technological advancements in the seafood industry have facilitated the development of a broad range of appealing ready-to-eat and ready-to-cook products, which have significantly altered consumer perceptions of seafood. These innovative product forms are attracting new consumer targets that avoid seafood due to preparation time or a general dislike for fresh fish products, particularly among younger consumers [3,4]. While some products, such as surimi or fish cakes, are clearly identified as mixtures of different species, others may disguise their composite nature under the appearance of higher-quality products like ‘fillets’, leading to misperceptions among consumers. The diffusion of these new seafood forms not only boosts industry profits but also increases risks to consumers and marine ecosystems. The assembly of mixed species in these products can facilitate illegal practices that compromise food safety, and promote fraudulent activities involving species substitution [5,6,7,8]. Indeed, such practices may pose health risks, particularly when allergenic species or specimens contaminated with harmful compounds, such as toxins or heavy metals, are introduced into the supply chain [9,10,11]. Furthermore, Illegal, Unreported, and Unregulated (IUU) fishing activities contribute to the overexploitation of fish stocks and the degradation of marine ecosystems, compounding the ecological and economic challenges faced by global fisheries [12,13]. Species substitution practices may also conflict with religious and ethical behaviors [14].

To date, European Union (EU) food labeling legislation, specifically Regulation (EU) No. 1169/2011 and Regulation (EU) No. 1379/2013, has focused on ensuring transparency, safety, and fairness in the seafood industry [15]. Regulation (EU) No. 1379/2013, in particular, encourages the use of molecular techniques to verify the identity of products. DNA-based methods have become popular for identifying seafood species due to the high resistance of target molecules during food processing [16,17]. In particular, DNA barcoding is a pivotal policy instrument for single-species identification [18,19]. However, traditional barcoding based on Sanger sequencing is limited to identifying the dominant species in complex food matrices [16]. High-throughput sequencing technologies (HTS), based on Next Generation Sequencing (NGS) platforms, enable the simultaneous sequencing of all DNA molecules present and, therefore, the identification of every species in a sample [20]. This approach, blending traditional DNA barcoding with NGS (DNA metabarcoding), has been primarily used in ecological studies [21,22,23] but has also proven effective in analyzing various food matrices. These include the floral composition of honey [24,25], ingredients in complex food products such as candies [26], meat [27,28,29,30], seafood [14,17,31,32], dairy products [29], spices [33,34], herbal supplements and teas [35,36,37], and pet food [38,39]. Despite its advantages, this approach has limitations that can significantly affect the results [40,41,42], particularly concerning the choice of molecular markers and primer pairs. These choices can influence PCR amplification, taxonomic profiles, and species-level resolution. To mitigate these biases, the use of multiple primer pairs from different molecular regions has been suggested for various applications, including biodiversity surveys, animal dietary analyses [40,41,42], mesozooplankton composition monitoring [43], identification of herbal teas and traditional medicines [36,44], and the authentication of mixed seafood products and canned tuna [32,45].

In this study, we assess the authenticity of processed single-species fish products using a multi-marker DNA metabarcoding approach. Specifically, two primer pairs targeting the mitochondrial cytochrome c oxidase I (COI) and 16S rRNA genes were sequenced using the Ion Torrent platform. The analysis aimed to (i) evaluate the universality of the primer pairs utilized, (ii) assess the discriminatory power at the species level of the two sequenced fragments, and (iii) compare molecular identifications with the list of ingredients reported on product labels.

## 2. Materials and Methods

### 2.1. Sampling

A total of 20 processed seafood samples (intact packages) from different brands and batches were collected from Italian supermarkets, including 5 burgers, 4 nuggets, 3 breaded fillets, 3 cutlets, 3 sticks, and 2 products labeled as “fish fantasy”. The labels indicated that these products were made up of one (18 samples) or two species. The samples were transported to the food safety laboratory in an insulated bag. The cooled containers were maintained at +4 °C and subsequently stored at −20 °C until the time of molecular analysis.

### 2.2. DNA Extraction, Amplification, and Sequencing

Genomic DNA was extracted and purified using the DNeasy Blood and Tissue Kit (QIAGEN, Hilden, Germany) in compliance with ISO 20813:2019 [46] and following the methodology described by Piredda et al., 2022 [14], at the Molecular Biology Laboratories of the Department of Veterinary Medicine at Bari. Briefly, under sterile conditions, three aliquots of 25 mg from each sample were collected and subsequently added to 180 μL of ATL lysis buffer and 20 μL Proteinase K (20 mg/mL). The aliquots were incubated at 56 °C for 1 h and 30 min. Then, 200 μL of AL buffer was added and incubated at 70 °C for 10 min. Subsequently, with the addition of 200 μL ethanol, the mixture was applied to DNeasy Mini-Columns (QIAGEN, Hilden, Germany). The DNA was adsorbed to the QIAamp silica-gel membrane by centrifugation at 6000× *g* for 60 s and subsequently washed with 500 μL of AW1 and 500 μL of AW2 washing buffers. Finally, the DNA was eluted using 60 μL of AE elution buffer (QIAGEN, Hilden, Germany). For each sample, the 3 aliquots were pooled and subjected to subsequent molecular analysis. To ensure the quality of the extraction process, positive controls (*Gadus chalcogrammus* Pure DNA Extract, Generon, San Prospero (MO), Italy) and negative extraction controls were included. The concentration and purity of the extracted DNA were assessed by measuring the absorbance ratios A260 nm/A280 nm and A260 nm/A230 nm using a NanoDrop^TM^ spectrophotometer (Thermo Fisher Scientific, Monza, Italy).

Amplifications and sequencing were performed at the laboratory of the Central Inspectorate for Fraud Repression and Quality Protection of Agri-Food Products and Foodstuffs-Ministry of Agriculture, Food Sovereignty and Forests (ICQRF-MASAF) located in Modena (Italy). The extracted DNA was amplified with the following primer pairs: 16sf-var 5′-CAAATTACGCTGTTATCCCTATGG-3′ and 16sr-var 5′-GACGAGAAGACCCTAATGAGCTTT-3′ [47] targeting a fragment of 148–209 bp of the 16S ribosomal RNA mitochondrial (16S) gene and FishCOIlbc 5′-CTCAACYAATCAYAAAGATATYGGCAC-3′ and Revshort1 5′-GGYATNACTATRAAGAAAATTATTAC-3′ [31], for the COI gene. These primers targeted fragments of 148–209 bp and 139 bp, respectively. After accurate assessment of dsDNA concentration using a Qubit^TM^ fluorimeter, amplification reactions were performed for each target fragment in a final volume of 50 μL, comprising 42 μL of Platinum™ PCR SuperMix High Fidelity (Thermo Fisher Scientific), 5 μL of the primer pool 2 μM, and 3 μL of DNA template 20–50 ng. The process used a QuantStudio™ 6 PRO PCR System (Thermo Fisher Scientific) with the following thermal profile: an initial denaturation at 95 °C for 3 min, followed by 30 cycles of denaturation at 95 °C for 30 s, annealing at 60 °C for 30 s (for the 16S gene) and 51 °C for 30 s (for the COI gene), and elongation at 68 °C for 2 min. Post-amplification, PCR products were purified using an Agencourt™ AMPure PCR purification kit (Beckman Coulter. Beverly, MA, USA) with a DynaMag™-96 Side Magnet (Thermo Fisher Scientific), following the manufacturer’s instructions. The integrity of the PCR products was verified by 1.5% (*w*/*v*) agarose gel electrophoresis stained with Green Gel Safe 10,000 × Nucleic Acid Stain (5 μL/100 mL; Thermo Fisher Scientific, Waltham, MA, USA). The purified products were then quantified using a Qubit™ fluorimeter (Thermo Fisher Scientific, Monza, Italy) and pooled in equimolar amounts for subsequent analysis.

The DNA barcode libraries for NGS were prepared from each amplicon pool using the Ion Plus Fragment Library Kit (Thermo Fisher, Monza, Italy), adhering to the manufacturer’s guidelines. All libraries were quantified, and their quality was assessed using a Qubit™ fluorimeter (Thermo Fisher Scientific, Monza, Italy). Libraries were diluted and pooled to achieve a 100 pM equimolar concentration for the template reaction. This process involved attaching DNA fragments to Ion Sphere Particles (ISPs) for clonal amplification via emulsion PCR, conducted using the Ion Chef™ System (Thermo Fisher, Monza, Italy). Following preparation, samples were sequenced on the Ion GeneStudio S5™ Prime sequencer (Thermo Fisher Scientific, Monza, Italy) utilizing an Ion 530 chip. The sequencing reads were analyzed using Torrent Suite^TM^ version 5.4 (Thermo Fisher Scientific. Monza, Italy).

### 2.3. Bioinformatics Analysis

After sequencing, BAM files containing nucleotide sequences in binary form were converted to *FASTQ* format using the SAMTOOLS Version 1.2 package [48]. For each *FASTQ* file, primers were removed; DNA reads were filtered by length and quality using Mothur v.1.48.0 [48]. The quality control settings included a maximum homopolymer length of 8 bp, no ambiguous bases allowed, and a read length between 80 and 250 bp. Reads were also screened for chimeras. Taxonomic assignments for representative Amplicon Sequence Variants (ASVs) were performed using the standalone BLAST+ suite [49,50] against a custom database including the 16S and COI mitochondrial sequences downloaded from GenBank (March 2023). Assignments with a similarity of less than 90%, indicative of potential low-quality reads or unknown taxa, were discarded. Sequences achieving 98–100% similarity were assigned at the species level [51], while those with less than 98% similarity were classified at the genus level. The raw sequence data were deposited in the Sequence Read Archive (SRA) under the BioProject database PRJNA 1136829.

### 2.4. Label Analysis and Mislabeling Assessment

Each product’s label was examined for compliance with Regulation (EU) No. 1169/2011, checking the commercial name, ingredient list, net quantity, storage instructions, expiration date, manufacturer information, nutritional declaration, and allergens. Molecular identifications were then compared with the species listed on the labels. Cases of mislabeling were identified if the species listed, either by scientific name or commercial designation, were not detected in the molecular analysis. When labels provided only the commercial designation, Annex I of the Italian MASAF Decree dated 22 September 2017 was referenced to determine the corresponding scientific names. This Decree was also used to verify the presence of taxa unmarketable in Italy.

## 3. Results and Discussion

In this study, we employed a multi-marker DNA metabarcoding approach to assess the species composition of processed seafood products that were presumably mono-species. Interestingly, the taxonomic profiles generated indicated that none of the products tested strictly adhered to a mono-species composition, and various species were either intentionally or accidentally included as ingredients. Furthermore, the analysis highlighted several limitations in the performance of the two primer pairs used, particularly in their ability to accurately trace products predominantly composed of Gadiformes species.

### 3.1. Molecular Identifications

The molecular analysis of 20 processed seafood samples using two DNA markers revealed the presence of 18 taxa spanning 2 classes (*Actinopteri*, *Asteroidea*), 5 orders (*Perciformes*, *Salmoniformes*, *Pleuronectiformes*, *Gadiformes*, *Forcipulatida*), 8 families, and 11 genera. The taxonomic profiles generated by the COI and 16S markers were only partially overlapping, which underscores the need for a multi-marker approach in metabarcoding studies for food authentication, as supported by recent research [29,32,36,45]. Differences between the taxonomic profiles derived from the two primer pairs can be attributed to variations in the universality of the primers and their discrimination power at the species level. For instance, approximately 67% of sequences in the COI dataset were taxonomically assigned to the genus *Gadus* sp., followed by *Merluccius* sp. (28.3%), with other taxa such as *Sander* lucioperca, *Arctogadus* glacialis, *Pollachius* sp., *Pleuronectes* platessa, and *Sebastes* sp. detected in smaller proportions (overall < 1.5%). The 16S dataset showed about 60% of sequences assigned to *Gadus* sp., followed by *Pleuronectidae* (31.1%), with additional taxa including *Salmo* salar, *Pollachius* virens, *Leptasterias* coei, *Limanda* aspera, and *Chelidonichthys* sp. (Perciformes) detected in lesser quantities (overall < 6%) (Supplementary Files S1).

**Figure 1:**
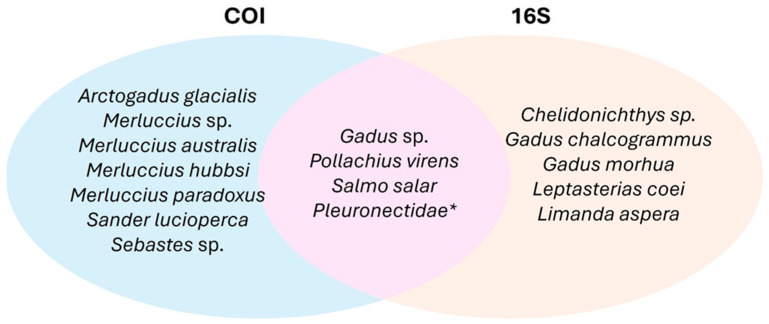
Venn diagram illustrating the shared and exclusive taxonomic identifications between the COI and 16S gene fragments. While Pleuronectidae is identified as a shared taxon, only the COI fragment achieved species-level identification. * Family detected by both markers but only COI was able to identify *Pleuronectes platessa* to species level.

This analysis not only confirmed four taxa shared between markers (*Gadus* sp., *Salmo* salar, *Pollachius* sp., and *Pleuronectidae*) but also identified seven taxa exclusive to the COI marker (*Arctogadus* glacialis, *Merluccius* sp., *Merluccius* australis, *Merluccius* hubbsi, *Merluccius* paradoxus, *Sander* lucioperca, *Sebastes* sp.) and five exclusive to the 16S marker (*Chelidonichthys* sp., *Gadus* chalcogrammus, *Gadus* morhua, *Leptasterias* coei, and *Limanda* aspera) (Figure 1).

Although both primer sets detected the presence of the genus *Gadus*, they differed in their ability to discriminate between species within the genus. The COI marker did not differentiate between *Gadus* chalcogrammus and *Gadus* morhua, whereas the 16S marker effectively discriminated between them, assigning 36% of sequences to *Gadus* chalcogrammus and 16% to *Gadus* morhua. Additionally, the genus *Merluccius* was only detected by the COI marker, which successfully identified the species *M.* australis, *M.* hubbsi, and *M.* paradoxus. However, four other species within this genus (i.e., *M.* productus, *M.* gayi, *M.* angustimanus, and *M.* bilinearis) were not discriminated by this marker.

These results, pertaining to two commercially significant genera (*Gadus* and *Merluccius*) in terms of economic value and global fisheries and trade, underscore the critical importance of primer selection during experimental design in metabarcoding assessments. Furthermore, although both markers detected the presence of the *Pleuronectidae* family, only the COI marker could identify *Pleuronectes* platessa at the species level, as detailed in Supplementary Files S1.

Despite employing two distinct primer pairs, the study encountered significant challenges in tracing the genus *Macruronus* (Order Gadiformes). The public molecular databases currently include sequences for only two species within this genus, *M.* novaezelandiae and *M.* magellanicus, but lack data for *M.* capensis. Consequently, in two samples (SM10 and SM11) where *Macruronus* capensis was declared on the label, its presence could not be verified because no reference sequences were available in public repositories. Moreover, the absence of matching sequences for the other two known species (*M.* novaezelandiae, M. magellanicus) suggests that both primer pairs were ineffective amplification for this genus. Interestingly, despite expectations based on label claim, no crustacean DNA was detected by either marker.

### 3.2. Label Analysis and Product Composition

The analysis of labels for all 20 processed seafood samples confirmed compliance with the mandatory requirements set by Regulation (EU) No. 1169/2011. Of these, 14 out of 20 labels (70%) voluntarily included both the commercial designation and the scientific name of the fish used. The remaining six labels (30%) provided only the commercial designation of the fish.

Based on our mislabeling criteria, which defines mislabeling as the absence of molecular identification of the species declared on the label, the comparison of molecular identifications obtained by both markers with the information on the labels revealed that each product contained the declared species, at least at the genus level. This finding indicates a generally high level of compliance with voluntary declarations (Table 1). However, 2 out of 20 samples (10%)—SM11 and SM23—were found to be mislabeled. In SM11, the non-compliance involved the absence of crustacean species listed under the commercial name “Gambero Indopacifico”. Such an outcome could be due to the actual absence of “Gambero Indopacifico” as an ingredient. Intriguingly, in sample SM23, labeled as breaded fillet of “Merluzzo d’Alaska”, *Gadus* chalcogrammus was entirely substituted with species from the *Merluccius* genus.

The observed substitution does not appear to be driven by economic motives, as hakes are generally considered higher-value species compared to Pollock [52]. Rather, the substitution may stem from confusion arising from the common names of these species, as both are referred to as “Merluzzi” in Italian. To mitigate such potential misunderstandings, which can occur among various intermediaries within the global supply chain, the adoption of Industry 4.0 technologies—such as artificial intelligence, blockchain, and big data—could enhance traceability “From Boat to Plate”. This integration promises to clarify product identities throughout the supply chain, thus supporting accurate labeling and compliance [52,53,54].

**Figure 2:**
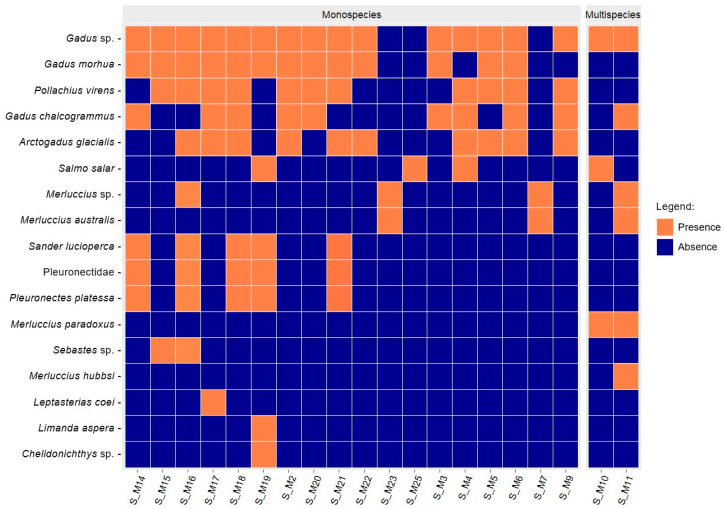
Heatmap of presence/absence based on the integration of molecular identifications obtained with COI and 16S fragments. Taxonomic identifications are reported at the species level where possible.

Although the products analyzed were labeled as containing one or two species, molecular analyses revealed that 95% of them were multi-species (Figure 2), containing species not listed in the ingredients. These unexpected species included commercially recognized fish such as *Pleuronectes platessa*, *Pollachius virens*, *Sander lucioperca*, *Merluccius australis*, *Sebastes* sp., *Limanda aspera*, and *Chelidonichthys* sp., but also species not listed in the latest Italian MASAF Decree (2017), such as *Arctogadus glacialis* and *Leptasterias coei*. The presence of these species could be economically motivated, as the intentional addition of fish discards and low-value fish is a known industry strategy to increase product weight and boost profits [10,14,54,55]. However, the presence of untraced seafood might also stem from illegal, unreported, and unregulated (IUU) fishing activities, raising serious ethical and ecological concerns [56]. Moreover, such practices can pose risks to human health through unintentional exposure to heavy metals, persistent organic pollutants, and microplastics [57].

On the other hand, accidental inclusion of species could occur due to contamination along the seafood supply chain, particularly when different raw materials are processed within the same facility [14]. An illustrative case was the detection of the inedible sea star species *Leptasterias coei* (Echinodermata: Asteroidea) in sample SM17, which was labeled and molecularly identified as containing *G. chalchogrammus* (Merluzzo d’Alaska). Interestingly, the geographical distribution of *Leptasterias coei* in the Northeast Pacific, specifically Alaska, provides indirect evidence of the harvesting region for *G. chalchogrammus*. Moreover, the accidental presence of this species highlights concerns about biodiversity loss associated with bottom trawling practices [58].

## 4. Conclusions

Overall, our results confirm that the metabarcoding approach is a critical and effective tool for seafood traceability. Integrating molecular profiles obtained using multiple primer pairs has enhanced species identification success in seafood products. While both primer pairs used in this study exhibited limitations in tracing Gadiformes—the taxa most commonly used in these products—the COI fragments, considered the gold standard for metazoan assessments [59], performed marginally better.

Given the importance of primer selection, an optimal strategy for thorough species characterization in seafood could involve pairing one primer pair designed to amplify and distinguish species listed on product labels (expected species) with another primer pair that offers broader taxonomic coverage to detect unexpected species.

Food fraud poses significant concerns for consumers, the food industry, and regulatory authorities, thus underscoring the need for innovative tools to ensure product authenticity. Although additional species detected in our study were not categorized under mislabeling criteria, our findings suggest that truly mono-species products are rare in processed seafood. Voluntary declarations on labels often present a truncated list of ingredients, potentially used by brands as a marketing tactic to appeal to consumers seeking authentic, safe, and wholesome products ostensibly made from a single species of fish [14]. The urgency for suitable verification tools is clear. For highly processed matrices, NGS approaches stand as the sole effective methods capable of tracing the full species composition of products. Therefore, further development is necessary, particularly in laboratory setups and bioinformatic pipelines, before these methodologies can be routinely employed in official controls by authorities or within the food industry [60]. However, their implementation will advance the “From Boat to Plate” strategy and combat fraudulent practices along the intricate fishery supply chain, in alignment with Regulation (EU) 2017/625.

## Figures and Tables

**Table 1:** Label details of processed seafood products alongside molecular identifications and instances of mislabeling detected using COI and 16S mitochondrial genes.

Sample ID	Type of Product	Commercial Designation	Scientific Name	Molecular Identification COI	Molecular Identification 16S	Molecular Identification COI_16S	Mislabeling COI_16S
SM10	Burger	Salmone and merluzzo sudafricano	*S. salar* ^a^, *Macruronus capensis or Merluccius paradoxus or Merluccius capensis*	***Salmo salar***: *Gadus* sp. ^c^: ***Merluccius paradoxus***	***Salmo salar***	*Gadus* sp.: ***Salmo salar***: *Merluccius paradoxus*	No
SM11	Burger	Merluzzo sudafricano and Gambero Indopacifico	*Macruronus capensis* ^a^ *o Merluccius paradoxus o Merluccius capensis* Crustaceans	*Merluccius* sp. ^b^: *Merluccius hubbsi*: ***Merluccius paradoxus***	*Gadus chalcogrammus*: *Gadus* sp. ^d^	*Merluccius hubbsi*: *Merluccius* sp.: *Gadus* sp.: *Gadus chalcogrammus*: ***Merluccius paradoxus***	Yes
SM14	Burger	Merluzzo Alaska	*G. chalcogrammus*	***Gadus* sp.** ^c^: *Sander lucioperca*: *Pleuronectes platessa*	*Pleuronectidae*: *Gadus* sp. ^d^: *Gadus morhua*: ***Gadus chalcogrammus***	*Gadus* sp.: ***Gadus chalcogrammus***: *Gadus morhua*: *Sander lucioperca*: *Pleuronectes platessa*	No
SM15	Fish fantasy	Merluzzo Alaska	*G. chalcogrammus*	***Gadus* sp.** ^c^: *Pollachius virens*: *Sebastes* sp.	*Pollachius virens*: ***Gadus* sp.** ^d^: *Gadus morhua*	*Gadus* sp.: *Pollachius virens*: *Gadus morhua*: *Pollachius virens*: *Sebastes* sp.	No
SM16	Sticks	Merluzzo Alaska	*G. chalcogrammus*	***Gadus* sp.** ^c^; *Sander lucioperca*: *Pleuronectes platessa*: *Arctogadus glacialis*: *Merluccius* sp.^b^: *Sebastes* sp.	*Pleuronectidae*: *Gadus morhua*: ***Gadus* sp.** ^d^	*Arctogadus glacialis*: *Gadus* sp.: *Merluccius* sp.: *Sander lucioperca*: *Pleuronectes platessa*: *Gadus morhua*: *Pleuronectidae*	No
SM17	Nuggets	Merluzzo Alaska	*G. chalcogrammus*	***Gadus* sp.** ^c^; *Arctogadus glacialis*; *Pollachius virens*	*Gadus* sp. ^d^: ***Gadus chalcogrammus***: *Leptasterias coei* ^e^: *Gadus morhua*	*Arctogadus glacialis*: *Gadus* sp.: *Pollachius virens*: *Gadus* sp.: *Gadus chalcogrammus*: *Gadus morhua*: *Leptasterias coei* ^e^	No
SM18	Cutlets	Merluzzo Alaska	*G. chalcogrammus*	***Gadus* sp.** ^c^: *Pleuronectes platessa*: *Pollachius virens*: *Sander lucioperca*: *Arctogadus glacialis*	*Pleuronectidae*: *Gadus* sp. ^d^: *Gadus morhua*: ***Gadus chalcogrammus***	*Gadus* sp.: *Pollachius virens*: *Sander lucioperca*: *Pleuronectes platessa*: *Gadus chalcogrammus*: *Gadus morhua*: *Pleuronectidae*: *Arctogadus glacialis*	No
SM19	Burger	Merluzzo Alaska	*G. chalcogrammus*	***Gadus* sp.** ^c^: *Pleuronectes platessa*: *Salmo salar*: *Sander luciorerca*	*Pleuronectidae*: *Chelidonichthys* sp.: *Limanda aspera*: ***Gadus* sp.** ^d^: *Gadus morhua*	***Gadus* sp.**: *Pleuronectes platessa*: *Salmo salar*: *Sander luciorerca*: *Gadus morhua*: *Pleuronectidae*: *Chelidonichthys* sp.: *Limanda aspera*	No
SM2	Braded fillet	Merluzzo Alaska	*G. chalcogrammus* ^a^	***Gadus* sp.** ^c^: *Arctogadus glacialis*	***Gadus* *chalcogrammus***: *Gadus* sp. ^d^: *Gadus morhua*	*Arctogadus glacialis*: *Gadus* sp.: ***Gadus chalcogrammus***	No
SM20	Fish fantasy	Merluzzo Alaska	*G. chalcogrammus*	***Gadus* sp.** ^c^: *Pollachius virens*	***Gadus* *chalcogrammus***: *Gadus* sp. ^d^: *Gadus morhua*: *Pollachius virens*	*Gadus* sp.: *Pollachius virens*: ***Gadus chalcogrammus***: *Gadus morhua*: *Pollachius virens*	No
SM21	Cutlets	Merluzzo Alaska	*G. chalcogrammus*	***Gadus* sp.** ^c^: *Pleuronectes platessa*: *Sander lucioperca*: *Pollachius virens*: *Arctogadus glacialis*	*Pleuronectidae*: *Gadus morhua*: ***Gadus* sp.** ^d^	***Gadus* sp.**: *Pollachius virens*: *Sander lucioperca*: *Pleuronectes platessa*: *Gadus morhua*: *Pleuronectidae*	No
SM22	Braded fillet	Merluzzo nordico	*G. morhua*	***Gadus* sp.** ^c^: *Arctogadus glacialis*	***Gadus* *morhua***: *Gadus* sp. ^d^;	*Arctogadus glacialis*: *Gadus* sp.: ***Gadus morhua***:	No
SM23	Braded fillet	Merluzzo Alaska	*G. chalcogrammus* ^a^	*Merluccius* sp. ^b^	N.A.	*Merluccius* sp.	Yes
SM25	Burger	Salmone	*S. salar*	***Salmo*** ***salar***	***Salmo*** ***salar***	***Salmo*** ***salar***	No
SM3	Nuggets	Merluzzo Alaska	*G. chalcogrammus*	***Gadus* sp.** ^c^	***Gadus* *chalcogrammus***: *Gadus* sp. ^d^: *Gadus morhua*	*Gadus* sp.: ***Gadus chalcogrammus***: *Gadus morhua*	No
SM4	Sticks	Pollak Alaska	*G. chalcogrammus*	***Gadus* sp.**: *Arctogadus glacialis*: *Pollachius virens*: *Salmo salar*	***Gadus* *chalcogrammus***: *Gadus* sp.^d^	*Arctogadus glacialis*: *Gadus* sp.: *Pollachius virens*: *Salmo salar*: ***Gadus chalcogrammus***	No
SM5	Nuggets	Merluzzo nordico	*G. morhua*	***Gadus* sp.** ^c^: *Arctogadus glacialis*	***Gadus* *morhua***: *Gadus* sp.^d^	*Arctogadus glacialis*: *Gadus* sp.: ***Gadus morhua***	No
SM6	Nuggets	Merluzzo Alaska	*G. chalcogrammus* ^a^	***Gadus* sp.** ^c^: *Arctogadus glacialis*	***Gadus* *chalcogrammus***: *Gadus* sp. ^d^: *Gadus morhua*	*Arctogadus glacialis*: *Gadus* sp.: ***Gadus chalcogrammus***: *Gadus morhua*	No
SM7	Cutlets	Merluzzo del Pacifico	*M. gayi*	***Merluccius* sp.** ^b^: *Merluccius australis*	N.A.	***Merluccius* *sp*.**^b^: *Merluccius australis*	No
SM9	Sticks	Merluzzo Alaska	*G. chalcogrammus* ^a^	***Gadus* sp.** ^c^	***Gadus* *chalcogrammus***: *Gadus* sp.^d^: *Pollachius virens*	*Gadus* sp.: ***Gadus chalcogrammus***: *Pollachius virens*	No

N.A.: not available. Species expected based on label information are highlighted in bold. ^a^ Species not declared on the label. The scientific name was extracted from Annex I of the Italian MASAF Decree dated 22 September 2017. ^b^ *Merluccius* sp. (COI) refers to the undistinguished species *M. productus*/*M. gayi*/*M. angustimanus*/*M. bilinearis*. ^c^ *Gadus* sp. (COI) refers to the undistinguished species *G. morhua*/*G. chalcogrammus.* ^d^ *Gadus* sp. (16S) refers to the undistinguished species *G. morhua*/*G. chalcogrammus.* ^e^ Species not listed in Annex I of the Italian MASAF Decree dated 22 September 2017.

## Data Availability

The original contributions presented in the study are included in the article/Supplementary Materials, further inquiries can be directed to the corresponding author.

## Supplementary Materials

The following supporting information can be downloaded at https://www.mdpi.com/article/10.3390/foods13152382/s1, Supplementary Files S1.
